# Mental Health and Proximal Stressors in Transgender Men and Women

**DOI:** 10.3390/jcm8030413

**Published:** 2019-03-25

**Authors:** Noelia Fernández-Rouco, Rodrigo J. Carcedo, Félix López, M. Begoña Orgaz

**Affiliations:** 1 Department of Education, Faculty of Education University of Cantabria, Av. de Los Castros s/n, 39005 Santander, Spain; fernandezrn@unican.es; 2 Department of Developmental and Educational Psychology, Faculty of Psychology, University of Salamanca, Av. Merced 109-131, 37005 Salamanca, Spain; flopez@usal.es (F.L.); borgaz@usal.es (M.B.O.)

**Keywords:** transgender, anxiety, depression, social loneliness, romantic loneliness

## Abstract

This paper explores the subjective perception of some personal and interpersonal aspects of the lives of transgender people and the relationship they have with their mental health. One hundred and twenty transgender people (60 men and 60 women) participated in semi-structured interviews. Following quantitative methodology, analysis highlighted that social loneliness is the main predictor of lower levels of mental health (anxiety and depression) for both genders and recognized romantic loneliness as the strongest factor among transgender men. In both cases, higher levels of loneliness were associated with lower levels of mental health. The results have guided us to improve institutional and social responses and have provided an opportunity to promote the mental health of transgender people.

## 1. Introduction

The mental health of transgender people is frequently disturbed in several spheres [1]. According to this, the Minority Stress Model asserts that mental health distress is often the result of a hostile or stressful social environment [2]. This model describes the processes by which sexual and gender minorities are subjected to minority stress: (a) distal or external stressors (environmental), such as exposure to discrimination and violence; (b) proximal interpersonal stressors such as feelings or expectations that external stressors will occur and the need to protect oneself from these external stressors; and (c) proximal personal stressors that reflect an internalization of negative attitudes and prejudice from society. Conversely, interactive and internalized proximal resilience is also possible, with internalization of positive self-image, use of adaptive coping skills and community attachments. Interactive and internalized proximal stressors are frequently described as distressing. The cumulative stressors can serve to overwhelm themselves and to lead to poor mental health outcomes [3].

Over the last decades, several studies have been focused on transgender people’s mental health and other personal and interpersonal variables (stressors) including self-esteem and body image [4], coping skills [5], social and emotional loneliness [6], sexual satisfaction [7] or anxiety and depression [8], yet there are no studies in Spain analysing how all these topics are able to explain the state of transgender people’s mental health.

This work focuses on internalized proximal stressors (self-esteem, body image and coping skills) and interpersonal ones (social and emotional loneliness and sexual satisfaction), as well as the associations occurring in transgender people’s mental health (anxiety and depression). To improve the empowerment and mental health of transgender people, proximal stressors (more modifiable taking into account personal aspects) need to be identified, which would provide both transgender people and professionals the opportunity to intervene.

### 1.1. Mental Health: Anxiety and Depression in Transgender People

The concepts of mental health and the specific nature of the relationship between anxiety and depression have been much debated. Research from the past decades has been reviewed to assess whether there is a quantitative or qualitative difference between anxiety and depression. Anxiety and depression syndromes have been studied both separately and combined to determine whether a quantitative or qualitative difference exists between them [9]. In the end, although there are several studies supporting comorbidity between anxiety and depression [10], they are commonly perceived as different; depressed disorders are characterized by a devaluation of self and negative attitudes toward the past and future, whereas anxiety disorders are marked by themes of danger and anticipated harm [11].

Although mental health problems may be self-limiting or may respond to self-help or to lay-help [12], delaying or avoiding formal care can result in problematic consequences. Too, the duration of untreated illness is associated with worsened outcomes in mental health problems such as major depressive and anxiety disorders [13]. The stigma resulting from a context in which power is exercised to the detriment of members of a social group [14], in this case, transgender people, includes such behaviours as labelling, separation, stereotype awareness and prejudice and discrimination. This stigma, along with mental health problems, is an important factor which prevents people from seeking help [15]. Additionally, this stigma plays an important role in limiting the opportunities and access to resources of transgender people in a number of critical domains (e.g., employment, healthcare, etc.), while continuously having a detrimental effect on their mental health [16]. A large body of literature points out that transgender people experience greater mental health problems, such as depression and anxiety [17,18] than do cisgender individuals (cisgender refers to those who are not transgender). Concretely, transgender people experience greater quantities of stressors from childhood which result in an increase of mental health problems such as depression and anxiety [19]. Transgender individuals, too, face a host of minority stressors specific to their sexual and gender minority identities. Viewed from a broader perspective, stigmatized people may be more susceptible to mental health problems due to the accumulation of stressors experienced over the course of a lifetime, as opposed to simply experiencing those stressors in isolated, discrete moments [18].

In addition, many community-based surveys have found that women (with no differences between cisgender and transgender), on average, experience depressed moods more frequently than men, as measured by self-report scales [20]. Women also self-report higher levels of anxiety [21]. Too, though the range of anxiety being studied varies, findings show that transgender men experience anxiety more frequently than transgender women [8,22,23].

### 1.2. Proximal Stressors in Transgender People

Transgender people have been found to face multiple difficulties and interpersonal challenges [24]. Forms of rejection from family and loved ones [25], low levels of self-esteem [26] and body image problems resulting from an attempt to reject those body parts that they do not identify with [27], are all examples of such challenges. Furthermore, although the association between transgender status and sexuality is commonly taken for granted and though research exists regarding improvements in sexual functioning after transition [28] and the importance of sexual life for humans in general [29], there is no substantial evidence pointing to sexual satisfaction in this population but rather to an unsatisfactory sex life [30]. In any case, the importance of social relations is not unique to the transgender population; humans are social beings who form attachments from the moment they are born [31]. They have a fundamental, adaptive need to belong [32]. Additionally, coping skills are vital to living a successful life and to maintaining a healthy mental health state [33,34]. Coping mechanisms, therefore, have been theorized to buffer the effects of mental health problems which result from stigmatization [2].

The impact that stressors have on both physical and mental health have been summarized in previous studies [3]. This literature, however, does not take gender into account when studying transgender status, nor have previous studies looked at transgender men or women individually. Finally, although certain stressors were studied both separately and jointly, no comprehensive studies yet exist in which proximal stressors are examined, including that of self-esteem, body image, coping skills, loneliness (social, family and romantic) and sexual satisfaction.

### 1.3. Associations Between Proximal Stressors and Mental Health for Transgender People

Much research exists linking different stressors to anxiety and depression. A large body of literature exists in which the relationship between self-esteem and depression is discussed. Furthermore, there is a growing body of longitudinal studies which indicate low levels of self-esteem predetermine depression; and correspondingly, people with high levels of self-esteem appear to have a lesser risk of suffering from depression [35]. In the same line, several theories postulate that a higher level of self-esteem serves as a buffer against anxiety [36]. This association was found within the transgender population as well, in relation to both anxiety and depression [37,38].

Body image is yet another factor that plays an important role in mental health [39]. Dissatisfaction with body image has been associated with an increase in mental health problems [40], a fact which holds true in the case of the transgender population [41]. The reinforcement of coping strategies, on the other hand, has proven effective in the management of issues encountered in the day to day, specifically in the prevention of problems related to mental health [42]. In fact, problem-focused coping predicted positive mental health outcomes among transgender youth [43] and the application of avoidant coping strategies during transitioning to manage gender-related stress has been associated with both depression and anxiety [8].

Interpersonal context has shown to be a major theme in the prevention or reduction of mental health problems. General loneliness was found to be an important variable for mental health [44]. Some authors have demonstrated that the emotional loneliness resulting from being cut-off from one’s family is the strongest variable related to issues in mental health [45]. A large percentage of transgender individuals experience family rejection, social isolation and loneliness, which can result in a number of negative issues including mental health problems [46].

Sexuality is also a central topic for human development [47]. Specifically, there is a reciprocal relationship between certain mental problems such as anxiety and depression and sex problems [48]. Some studies in which other excluded populations were subjects, discovered that sexual satisfaction predicts positive mental health [49,50,51,52]. In terms of the transgender population, most studies which investigate sexual function are focused only on post-surgical outcomes [53].

Previous work studies the relationship that exists between different stressors and mental health but does not take into account the role that gender may play in this relationship owing to the fact that men and women are usually studied together [54], nor are the ways in which gender could affect the associations between stressors and mental health yet determined.

In summary, existing research has demonstrated that self-esteem, body image, coping skills, loneliness and sexual satisfaction are predictors of depression and anxiety. However, existing studies have not yet examined the relationships that exist between each of these variables and how gender moderates these relationships. The purpose of this study, therefore, is to examine the pattern of connections among each of these variables as they relate to transgender individuals, both transgender men and women. The current study aims to investigate (1) whether higher levels of self-esteem, body image, proactive coping skills, sexual satisfaction and lower levels of loneliness will be associated to better mental health and (2) whether differences exist between men and women.

## 2. Experimental Section

### 2.1. Participants

The sample consisted of 120 transgender people residing within Spain (93.3% Spanish and 6.7% foreigners, all from South America), 60 men (female-to-male) and 60 women (male-to-female). Participants were recruited in different cities and villages by this article’s authors. Contact was made via phone call or emails sent to people in LGTB or Transgender non-profit organizations and internet forums on websites aimed at LGTB or transgender information. The age range of the sample was 18 to 63 years old (M = 33.8; S.D. = 10.1). Of the participants, 19.1% had primary studies, 15% finished secondary school, 38.3% finished professional training and 27.5% finished university. We selected participants while maintaining a balanced number of men and women in three different reassignment moments (i.e., persons who assumed gender without any hormonal or surgery treatment, persons in hormonal treatment, persons in surgery reassignment process and persons who fully reassigned their sex). After stratifying by gender and reassignment moments, they were selected under a “snowball” sampling scheme [55].

### 2.2. Procedure

The people who responded positively to the recruitment method were given a standard description of the study and were evaluated for their eligibility to participate which consisted of the following criteria: individuals had to identify themselves as exclusively transgender at the time of the interview, did not have any mental health problem diagnosis or current state that impede to answer accurately to an interview (e.g., schizophrenia or being under the influence of drugs, etc.), expressed a consistent desire to have reassignment surgery and were 18 years of age or older. Eligible participants who expressed an interest in participating in the study were interviewed in-person at a location of their choosing (e.g., home, cafeteria, etc.). Individuals participating in the study did so voluntarily and there were no incentives in exchange for participation. The study was conducted in Spanish.

Face to face interviews lasting about 90 min were conducted in which each participant was orally asked all the questions in order to assure that everything was fully understood, taking into account the modest educational level of a considerable percentage of participants. First author of this paper introduced herself as member of the university staff and expressed our interest in the experiences of transgender people. Only upon establishing rapport, informing participants that they were free to leave the study whenever they wished and that their participation was confidential and voluntary and explicitly obtaining informed consent, did interviews commence. Upholding these ethical standards is vital for the collection of good quality data. The Good Practice Manual for Research of CSIC (2011) was followed regarding ethical standards [56]. In addition, this study respected the norms of the Declaration of Helsinki.

### 2.3. Measures

#### 2.3.1. Predictor Variables

Self-esteem. The instrument used was the Tennessee Self-Concept Scale 2nd Edition (TSCS:2) developed by Fitts and Warren as a review of Tennessee Self-Concept Scale [57,58]. The complete scale consists of 82 statements. The items are classified into three dimensions: (1) identity and self-concept: how does the individual see him/herself (30 items); (2) self-satisfaction or self-esteem: how does the individual accept him/herself (30 items); (3) self-behaviour: how does the individual behave towards him/herself (30 items). The short form is used with the first 20 questions and gives an indication of whether a person tends to see him/herself as generally positive and consistent or negative and variable. Scores from 1 (always false) to 5 (always true) are used, with higher scores reflecting higher levels of self-esteem. This instrument was chosen because it is standardized, easy to administer and has presented a good validity showing high correlations with other self-esteem scales [57]. Cronbach’s alpha in this study was 0.83.

Body Image. The Body Image Scale [59] was used. A higher score indicates higher levels of dissatisfaction. On the 14-item Appraisal of Appearance Inventory (AAI), three independent observers (the diagnostician, a nurse from the gender team and the researcher) rated their subjective appraisal of the appearance of the subject on a 5-point scale of femininity/masculinity. Higher scores indicate higher levels of incompatibility with the appearance of the new gender. Cronbach’s alpha in this study was 0.93.

Coping Skills. The Coping Skills Scale summarizes the dimensions described by Lazarus and Folkman [60,61]. It is a multidimensional instrument that assesses active coping, social support coping, avoidant coping cognitive passivity and repression and avoidant coping behaviour or refusal.

Certain items were eliminated from the scale for use in this study as they were not considered to be adequate indicators of the coping strategy. The scale has been adapted according to the characteristics of the participating sample and by combining the two avoidance coping subscales into one. The response format, however, was not altered: a scale from 1 (I have never faced a situation like that) to 4 (I have come into contact with a situation like that many times) was applied. An exploratory factor analysis was conducted in order to pinpoint these modifications, yielding five factors (66.95% variance explained) that ultimately were grouped into three factors to rule out the items in our sample did not indicate the use of the strategy for coping in the original scale: active coping strategy, coping strategies and social support avoidant coping strategy, which account for 60.96% of the variance.

In our study, internal consistency for the subscale of social support coping corrected (7,8,13,17) was an alpha of 0.70 for active coping subscale corrected (1,6,9,12) alpha was of 0.75 and avoidant coping subscale corrected (2,3,4,5,14) was 0.77.

Social and emotional loneliness. The short version of the Social and Emotional Loneliness Scale for Adults (SELSA-S) was used to measure both types of loneliness [45]. In fact, SELSA-S consists of three subscales labelled (a) social loneliness, (b) family-emotional loneliness and (c) romantic-emotional loneliness. Participants rated 15 items, 5 of every subscale. Items were rated on a 7-point Likert-type scale that ranged from 1 (strongly disagree) to 7 (strongly agree). The total score of every subscale was obtained by summing up the items, with possible scores ranging from 7 to 35. There is no total score for loneliness because this measure comes from a multidimensional perspective of loneliness. In this study Cronbach’s alpha was 0.83 for family-emotional, 0.77 for social and 0.74 for romantic-emotional.

Sexual satisfaction. The subscale of sexual satisfaction of the Multidimensional Sexual Self-Concept Questionnaire (MSSCQ) was used to measure this aspect [7]. A total 5 of 5 items were scored on a 7-point Likert-type scale (expanding upon the original 5-point Likert-type scale) ranging from 1 (not at all characteristic of me) to 7 (very characteristic of me) comparable to a SELSA scale. Alpha was 0.96 and 0.95 in this study.

#### 2.3.2. Moderator Variable

Gender was recorded as 0 for transgender women and 1 for transgender men.

#### 2.3.3. Outcome Variables

The Anxiety and Depression subscales of The Symptom Checklist of Derogatis (SCL-90-R) were used to assess anxiety and depression [62]. Twenty-three items were scored, ten items for anxiety and thirteen for depression. For each item the person was asked to rate severity of depression experienced over the past week. Responses were scored on a five-point scale ranging from (1) not at all to (5) extremely. Cronbach’s alpha was 0.92 for anxiety and 0.94 for depression,

For all the scales and subscales, a total score was obtained by adding up the individual scores and dividing them by the number of items answered.

### 2.4. Analysis Strategy

As the method of obtaining data was the interview method, no missing data was obtained; all of the participants answered every question. After data curation, statistical techniques were used to process the data using descriptive, Pearson correlations and hierarchical regression analysis with the IBM SPSS 22 package (IBM, Armonk, NY, USA). Firstly, pertinent analyses were carried out to verify the reliability, normality, independence and homoscedasticity assumptions using Cronbach’s alpha, Kolmogorov-Smirnov test and Q-Q plots, the collinearity statistics (tolerance index and variance inflated factor—VIF and the Breusch-Pagan test respectively. Secondly, independent samples *t*-test were used to assess the statistical significance of gender differences. Thirdly, Pearson bivariate correlations were used to explore the associations between men’s and women’s mental health and stressors. Fourthly, hierarchical multiple linear regression analysis was used to study the moderating effect of gender on the criterion variables (anxiety and depression). Before computing these, the assumptions of the presence of normality, linearity and homoscedasticity, along with the absence of multicollinearity were tested. Predictors were entered into the first step (main effects) and interactions between gender and predictors were entered in the second step (interactions between gender and those predictor variables that showed a different association seem to have responded in a different way in relation to the gender of the participants). When an interaction is significant, two separate regression models for each level of the moderator were conducted. Alpha level of 0.05 was used. Finally, power analysis was obtained using the G*Power program [63] and heteroscedasticity between the predictor and the criterion variable was run through the macro Heteroskedasticity SPSS [64].

## 3. Results

The Cronbach’s alpha showed a good reliability and the residual variance was constant with normality distribution.

All the predictors showed a linear relationship with anxiety and depression as it was observed in the scatterplot of the standardized residuals with the standardized predicted values. Q-Q plots and the level of significance obtained when applying the Kolmogorov-Smirnov test (up to 0.05) showed a good normality. When testing multicollinearity, the tolerance index values for the studied variables were up to 0.78 for anxiety and 0.69 for depression, which indicated the independence of the contributions of the predictor variables, producing variance inflated factor (VIF) scores lower than 10 for all the predictors. Finally, heteroscedasticity was an accomplished assumption because Breusch-Pagan (LM = 9.87; *p* = 0.20 for anxiety and LM = 3.05; *p* = 0.96 for depression) test was not found significant.

### 3.1. Gender Differences in Proximal and Mental Health Variables

Descriptive statistics of predictor and outcome variables are displayed in Table 1 for transgender men and women respectively. To examine whether there are mean differences based on the study variables, *t*-tests for independent samples were conducted. Differences in anxiety, body image, social loneliness and sexual satisfaction were found. Transgender women showed higher levels of anxiety, social loneliness and sexual satisfaction and a poorer body image.

### 3.2. Proximal Aspects and Mental Health for Transgender Men and Women

Bivariate correlations of interpersonal variables with anxiety and depression for both men and women, are shown in Table 2. All the stressors were associated with anxiety and depression except for the case of active and social support coping strategies which were not significantly correlated with anxiety. Similarly, high correlations were observed between anxiety and depression.

To identify whether associations of stressors with anxiety and depression varied by gender, two separate bivariate correlational analyses were conducted for transgender men and women (see Table 3). Regarding the correlation between the stressors and anxiety, men showed higher associations for family loneliness, whereas women showed higher associations for body image and social loneliness. With respect to the correlations between stressors and depression, men showed higher correlations for family and social loneliness, whereas women presented higher correlations for body image and avoidant coping strategies, interestingly not showing significant correlations with loneliness. Some correlations differ for men and women: anxiety and self-esteem, body image, avoidant coping strategy and romantic loneliness, depression and active coping strategy, social support coping strategy and romantic loneliness. These variables would be entered in the regression models as interactions with gender (see Table 3).

### 3.3. Proximal Stressors as Predictors of Mental Health (Anxiety and Depression)

To test the effects of all predictor variables on symptoms of anxiety and depression, taking into account the role of gender variable in those relationships (moderating effect), two hierarchical multiple regression analysis were conducted using the two-step model with two steps of independent variables.

**Figure 1:**
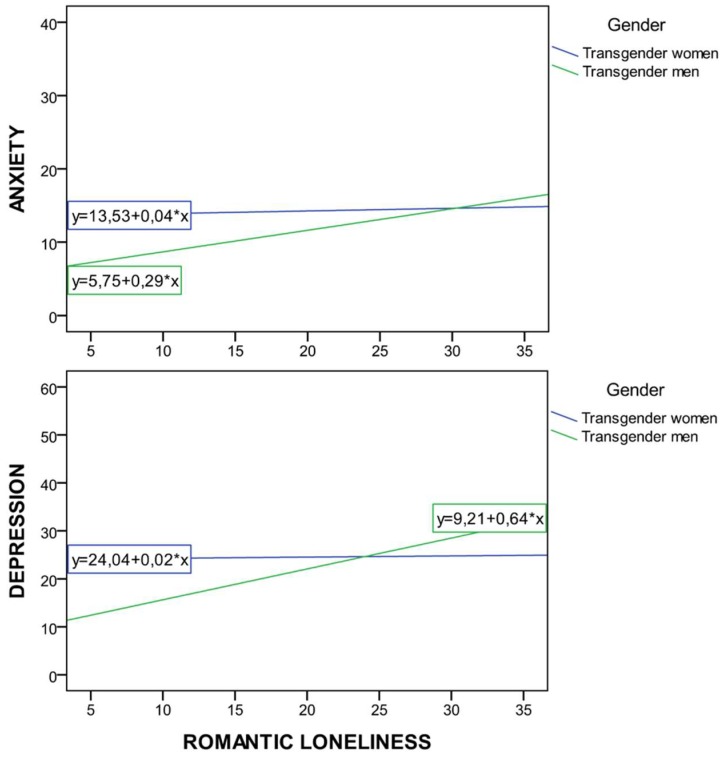
Romantic loneliness × gender interaction effect on mental health: (**a**) Depression; (**b**) Anxiety.

Taking into account anxiety as a criterion variable, the main effects model was significant (F (1, 118) = 36.87, *p* < 0.001). This model accounted for 23% of the variance of anxiety. In order to the study the moderating effect of gender, the interaction of each predictor with gender was also included in a second step (i.e., self-esteem, body image, avoidant coping strategy and romantic loneliness). The interactions model produced an increment of 4% variance and romantic loneliness × gender interaction was found to be significant. Hence, the interaction effects model was selected to explain anxiety (F (1, 117) = 7.79, *p* < 0.01) and its observed power was 0.85. In this sense, the predictor found to be significant for both genders was social loneliness and the predictor found only for men was romantic loneliness. Therefore, higher scores in social loneliness were associated with higher levels of anxiety for transgender men and women and higher scores in romantic loneliness were associated with higher levels of anxiety only for transgender men (F (1, 58) = 32.77, B = −0.78, *p* < 0.001) (see Table 4 and Figure 1).

Regarding the regression analysis conducted to explain depression, the main effects model was also significant (F (1, 116) = 12.15, *p* < 0.001). This model accounted for 53% of the variance of depression. The interaction of each predictor with gender was also included in a second step (i.e., active coping strategy, social support strategy and romantic loneliness) to study the moderating effect of gender. The interactions model produced an increment of 5% variance and romantic loneliness × gender interaction was found to be significant. Hence, the interaction effects model was selected to explain depression (F (1, 115) = 10.84, *p* < 0.001) and its observed power was 0.99. In this sense, the predictor found to be significant for both genders was social loneliness, avoidant coping strategy and body image and the predictor found only for men was romantic loneliness. Therefore, higher scores in social loneliness, avoidant coping strategy and a poor body image was associated with higher levels of depression for transgender men and women, and higher scores in romantic loneliness explained higher levels of depression for transgender men (F (1, 55) = 6.31, B = −0.49, *p* < 0.05) (see Table 4 and Figure 1).

## 4. Discussion

The study aimed to investigate the situation and the relationship between proximal stressors and mental health capacity among transgender adults in Spain. Several significant differences were found in some stressors and in the mental health of both men and women. Specifically, transgender women were found to have higher levels of anxiety, poorer body image, higher social loneliness and higher sexual satisfaction, similar to the results found in previous literature [65,66]. Women have been particularly stigmatized because by transitioning from male to female and deviating from their expected gender role, prior social status is lost [1,67]. Perhaps women show greater sexual satisfaction due to the fact that their sexual life is a private sphere, one that is not publicly visible and, unlike other contexts such as social relationships, it is a realm in which they can experience more freedom. There is no literature on this subject but it has become a topic of special interest for future research.

On the subject of mental health, anxiety and depression typically occur simultaneously [68], a fact which holds true for transgender people as well [69], although they are commonly accepted as separate concepts [70]. Considering the association between proximal stressors and mental health, this study investigated the effects of proximal personal (self-esteem, body image and coping skills) and proximal interpersonal (social, family and romantic loneliness and sexual satisfaction) stressors on transgender men’s and women’s psychological health (anxiety and depression). Identifying which are the most important predictors and how to minimize them would be a useful tool for the design of future clinical and research interventions. The findings are consistent with previous research in that several proximal stressors were found to be associated with poor mental health among transgender people [8,46,71].

With respect to the ways in which these stressors are associated with poor mental health, social loneliness accounts for anxiety in both men and women, whereas romantic loneliness only accounts for it in men. Additionally, depression is accounted for by the level of social loneliness, body image and the use of avoidant coping skills in both men and women, though it is only accounted for by romantic loneliness, again, in in the case of men. Gender differences, therefore, are only significant in the case of romantic loneliness. These results substantiate previous findings regarding these variables and that of psychological health in different populations. In fact, there is empirical evidence regarding the fact that loneliness anticipates anxiety and depression [44,72,73,74,75]. There are no previous studies, however, concerning the role of gender in regard to romantic loneliness.

On the other hand, coping skills also play a role in one’s mental health. The use of ineffective coping skills can either hinder or promote anxiety and depression [33]. The coping strategy of avoidance, which has shown to be ineffective in resolving complex life circumstances, causes people to experience significant levels of distress [76]. Discomfort with body and high desire of reassignment between the participants (at different levels) is common among members of our sample, related with a poor mental health [27].

For the results of our study to be accurately interpreted, certain limitations must be considered. First, the measures used in the study were all self-reported, a factor that may be associated to higher levels of response bias. Nevertheless, self-reported measures are an effective method in which to assess mental health [77]. Second, the study used a cross-sectional design which does not allow for understanding causal pathways. Nevertheless, the study contributes to our understanding of the significant association between proximal stressors and mental health, taking into account the moderating effect of gender. Third, the use of convenience sampling limits the generalizability of the findings, although it allowed us to access people in different situations. Fourth, the bidirectionality of the relationship between some of the stressors and the mental health can be considered a limitation. This issue is partially ameliorated by the fact that the outcome variables had a timeframe that was more proximal to the reporting period, whereas the independent variables had more distal timeframes, meaning that a larger body of literature exists in which proximal stressors foresee issues related to mental health. Finally, no other situations of disadvantage linked to mental health (socioeconomic status, culture, ethnicity, etc.), that could potentially have affected what was found in the analysis have been studied. Thus, in addition to transgender experience, gender was included as an important variable to be considered.

Future research should delve deeper, including looking into distal stressors and other mental health indicators. It could be interesting as well to separate those who have long-term mental health issues and those who do not. In this way, data collection and research projects are possible not only in the short-term but long-term as well, which would then allow for a more developmental perspective. Finally, qualitative research would allow for better understanding of subjective experiences in relation to stressors and mental health. All these research suggestions could be useful for a better understanding of the transgender experience.

Finally, notwithstanding the mentioned limitations and future research suggestions, the current study contributes to the literature on the subject by (1) exploring proximal personal and proximal interpersonal stressors and mental health in the transgender population, as well as differentiating between men and women; (2) highlighting the relationship between proximal stressors and mental health in this population; and (3) emphasizing the role of gender as a moderator of the relationship between stressors, specifically romantic loneliness and mental health. These contributions could lead to professional intervention which would promote the mental health of transgender people. Based on our results, interventions looking to reduce social loneliness, avoidant coping strategies, poor body image and romantic loneliness (among men) would be compelling, as would the impact each has on transgender individuals’ mental health. Practitioners should to be aware the importance of relationships and the impact of loneliness on in transgender’s mental health. Promoting a good relational network, both friendships and romantic partners, has always to be considered in any intervention with this population. In fact, working on social meaningful connections would buffer other feelings of loneliness, such as romantic loneliness. In this case, this seems to be especially important for transgender men in the context of romantic relationships.

Additionally, it is known that this population lives in stressful an environment due to different situations such as stigma, transphobia, and/or violence [1]. All these circumstances may promote the utilization of avoidant coping strategies in order to protect themselves from distress. However, as we have observed in this study, the use of these strategies may individuals be more prone to depression.

Finally, practitioners should focus on individuals’ body image. This is an important aspect in order to prevent depression. Developing an accurate evaluation and intervention and also working on individuals’ context to prevent from discrimination due to body image are important elements to be considered.

## Figures and Tables

**Table 1:** Descriptive statistics for men and women in predictor and outcome variables.

N = 120	Answer Range	Mean		SD		*t*	*p*
		Tr. Men	Tr. Women	Tr. Men	Tr. Women		
Anxiety	1–5	1.13	1.43	0.78	0.84	1.92	<0.05
Depression	1–5	1.65	1.89	0.94	0.98		
Body Image	1–5	2.81	3.09	0.58	0.72	2.35	<0.05
Self-esteem	1–5	3.42	3.44	0.54	0.53		
Active coping	1–4	2.74	2.79	0.48	0.48		
Soc. support coping	1–4	2.59	2.52	0.58	0.72		
Avoidant coping	1–4	2.17	2.25	0.46	0.50		
Social loneliness	1–7	3.30	3.54	1.39	1.45		
Family loneliness	1–7	3.78	3.54	1.67	1.77		
Romantic loneliness	1–7	3.82	4.39	1.55	1.39	2.13	<0.05
Sexual satisfaction	1–5	2.53	2.91	0.99	1.10	2.01	<0.05

**Table 2:** Bivariate correlations for all the sample (men and women together).

	1.	2.	3.	4.	5.	6.	7.	8.	9.	10.	11.
1. Anxiety		0.75 *	−0.29 **	−0.22 *	−0.09	−0.06	0.29 **	0.40 **	0.48 **	0.19 *	−0.19 *
2. Depression			−0.53 **	−0.45 **	−0.26 **	−0.25 **	0.58 **	0.50 **	0.59 **	0.23 **	−0.41 **
3. Body image				0.41 **	0.18 *	0.20 *	−0.46 **	−0.43 **	−0.35 **	−0.17	0.51 **
4. Self esteem					0.51 **	0.25 **	−0.36 **	−0.57 **	−0.54 **	−0.23 **	0.33 **
5. Active cop.						0.32 **	−0.33 **	−0.35 **	−0.43 **	−0.10	0.24 **
6. Soc. supp. cop.							−0.24 **	−0.18 *	−0.27 **	0.02	−0.03
7. Avoidant cop.								0.33 **	0.39 **	0.17	−0.33 **
8. Fam. lonel.									0.69 **	0.12	−0.19 *
9. Soc. lonel.										0.31 **	−0.26 **
10. Rom. lonel.											−0.38 **
11. Sex. satisfact.											

* *p* < 0.05; ** *p* < 0.01.

**Table 3:** Bivariate correlations for men and women separately (transgender men above the diagonal and transgender women below the diagonal).

	1.	2.	3.	4.	5.	6.	7.	8.	9.	10.	11.
1. Anxiety	1	0.73 **	−0.22	−0.26 *	−0.01	−0.08	0.23	0.47 **	0.52	0.29 *	−0.22
2. Depression	0.77 **	1	−0.51 **	−0.56 **	−0.20	−0.28 *	0.52 **	0.61 **	0.69 **	0.40 **	−0.47 **
3. Body image	−0.44 **	−0.63 **	1	0.34 **	0.04	0.15	−0.33 **	−0.31 *	−0.21	−0.24	0.57 **
4. Self esteem	−0.19	−0.36 **	0.48 **	1	0.49 **	0.22	−0.36 **	−0.62 **	−0.65 **	−0.32 *	0.36 **
5. Active cop.	−0.17	−0.33**	0.29 *	0.53 **	1	0.35 **	−0.25	−0.37 **	−0.44 **	−0.01	0.12
6. Soc. supp. cop.	−0.03	−0.21	0.26 *	0.28 *	0.29 *	1	−0.31 *	−0.18	−0.19	−0.01	0.02
7. Avoidant cop.	0.34 **	0.63 **	−0.60 **	−0.37 **	−0.42 **	−0.18	1	0.24	0.40 **	0.36 **	−0.44 **
8. Fam. lonel.	0.38 **	0.43 **	−0.51 **	−0.52 **	−0.33 **	−0.20	0.43 **	1	0.71 **	0.16	−0.17
9. Soc. lonel.	0.44 **	0.49 **	−0.51 **	−0.44 **	−0.43 **	−0.32 *	0.38 **	0.69 **	1	0.41 **	−0.35 **
10. Rom. lonel.	0.03	0.01	0.20	−0.15	−0.23	0.08	−0.05	0.11	0.19	1	−0.47 **
11. Sex. satisfact.	−0.24	−0.43 **	0.44 **	0.32 *	0.36 **	−0.06	−0.26 *	−0.19	−0.23	−0.39 **	1

* *p* < 0.05; ** *p* < 0.01.

**Table 4:** Hierarchical multiple regression analysis with anxiety and depression as dependent variables.

Anxiety						Depression					
Predictor Variables	Model 1		Model 2		95% CI (LL, UL)	Predictor Variables	Model 1		Model 2		95% CI (LL, UL)
	(B)	SE B	(B)	SE B			(B)	SE B	(B)	SE B	
Step 1: Proximal Stressors:						Step 1: Proximal Stressors:					
Self-esteem	0.06	0.09	0.04	0.07	(−0.05, 0.33)	Self-esteem	0.05	−0.18	−0.05	−0.03	(−0.25, 0.14)
Body image	−0.14	−0.11	−0.22	−0.19	(−0.19, 0.04)	Body image	−0.25 ***	−0.35	−0.31 ***	−0.23	(−0.24, −0.05)
Avoidant coping	0.12	0.10	0.13	0.10	(−0.36, 0.62)	Active coping	0.07	−0.02	0.01	0.02	(−0.24, 1.23)
Family loneliness	0.13	0.09	0.17	0.13	(−0.12, 0.32)	Soc. support coping	−0.03	−0.10	−0.02	−0.05	(−0.97, 0.31)
Social loneliness	0.56 ***	0.50	0.51 ***	0.45	(0.33, 0.68)	Avoidant coping	0.32 ***	0.40	0.33 ***	0.30	(0.63, 1.77)
Rom. loneliness	0.04	0.04	−0.01	−0.01	(−0.29, 0.10)	Family loneliness	0.13	0.16	0.06	0.07	(−0.14, 0.40)
Sex. satisfaction	−0.06	−0.07	−0.12	−0.13	(−0.47, 0.12)	Social loneliness	0.37 ***	0.60	0.26 ***	0.27	(0.21, 0.71)
						Rom. loneliness	0.02	0.05	−0.06	−0.12	(−0.34, 0.13)
						Sex. satisfaction	−0.11	−0.28	−0.13	−0.15	(−0.67, −0.03)
Moderator						Moderator					
Gender	−0.14	−0.13	0.49	0.51	(−5.80, −0.31)	Gender	−0.14	−0.07	0.39	0.33	(−6.09, −0.65)
Step 2: Interaction model:						Step 2: Interaction model:					
Self−esteem × Gend.			0.48	0.50	(−0.26, 0.25)	Active cop. × Gend.			0.10	0.14	(−1.9, 1.35)
Body image × Gend.			0.19	0.21	(−0.11, 0.20)	Soc. sup. cop. × Gend.			−0.20	−0.24	(−1.80, 0.68)
Avoidant cop. × Gend.			0.34	0.35	(−0.84, 1.11)	Rom. lonel × Gend.			−0.58 ***	−0.58	(−0.95, −0.20)
Rom. lonel. × Gend.			−0.46 ***	−0.22	(−0.26, −0.04)						
*R* ^2^	0.24		0.29			*R* ^2^	0.54		0.58		
*∆R^2^*	0.24 ***		0.05 **			*∆R^2^*	0.05 ***		0.04 ***		

** *p* < 0.01; *** *p* < 0.001/LL = Lower Level; UL = Upper Level.

## References

[1] Fernández-Rouco N., Carcedo R.J., Yeadon-Lee T. (2018). Transgender Identities, Pressures and Social Policy: A Study Carried Out in Spain. J. Homosex..

[2] Meyer I.H. (2003). Prejudice, social stress and mental health in lesbian, gay and bisexual populations: Conceptual issues and research evidence. Psychol. Bull..

[3] Hendricks M.L., Testa R.J. (2012). A conceptual framework for clinical work with transgender and gender nonconforming clients: An adaptation of the Minority Stress Model. Prof. Psychol. Res. Pract..

[4] Marone P., Iacoella S., Cecchini M., Rabean A. (1998). An Experimental Study of Body Image and Perception in Gender Identity Disorders. Int. J. Transgend..

[5] Diener E., Suh E., Oishi S. (1997). Recent findings on subjective Wellbeing. Indian J. Clin. Psychol..

[6] Van Tilburg T., Havens B., de Jong Gierveld J. (2004). Loneliness among older adults in the Netherlands, Italy and Canada: A multifaceted comparison. Can. J. Aging..

[7] Snell W.E., Davis C.M., Yarber W.L., Bauserman R., Schreer G., Davis S.L. (1995). The Multidimensional Sexual Self-Concept Questionnaire. Handbook of Sexuality Related Measures.

[8] Budge S.L., Adelson J.L., Howard K.A. (2013). Anxiety and depression in transgender individuals: The roles of transition status, loss, social support and coping. J. Consult. Clin. Psychol..

[9] Stavrakaki C., Vargo B. (1986). The relationship of anxiety and depression: A review of the literature. Br. J. Psychiatry.

[10] Gorman J.M. (1996). Comorbid depression and anxiety spectrum disorders. Depress. Anxiety.

[11] Tanaka E., Sakamoto S., Kijima N., Kitamura T. (1998). Different personalities between depression and anxiety. J. Clin. Psychol..

[12] Oliver M.I., Pearson N., Coe N., Gunnell D. (2005). Help-seeking behaviour in men and women with common mental health problems: Cross-sectional study. Br. J. Psychiatry.

[13] Dell’Osso B., Glick I.D., Baldwin D.S., Altamura A.C. (2013). Can long-term outcomes be improved by shortening the duration of untreated illness in psychiatric disorders: A conceptual framework. Psychopathology.

[14] Link B., Phelan J.C. (2001). Conceptualizing stigma. Am. Rev. Sociol..

[15] Clement S., Schauman O., Graham T., Maggioni F., Evans-Lacko S., Bezborodovs N., Morgan C., Rüsch N., Brown J.S., Thornicroft G. (2015). What is the impact of mental health-related stigma on help-seeking? A systematic review of quantitative and qualitative studies. Psychol. Med..

[16] Hughto J.M.W., Reisner S.L., Pachankis J.E. (2015). Transgender stigma and health: A critical review of stigma determinants, mechanisms and interventions. Sol. Sci. Med..

[17] Arcelus J. (2015). Non-Suicidal Self Injury in Transsexualism: Associations with Psychological Symptoms, Victimization, Interpersonal Functioning and Perceived Social Support. J. Sex. Med..

[18] Mustanski B., Andrews R., Puckett J.A. (2016). The effects of cumulative victimization on mental health among lesbian, gay, bisexual and transgender adolescents and young adults. Am. J. Public Health Res..

[19] Roberts A.L., Rosario M., Slopen N., Calzo J.P., Austin S.B. (2013). Childhood gender nonconformity, bullying victimization and depressive symptoms across adolescence and early adulthood: An 11-year longitudinal study. J. Am. Acad. Child Adolesc. Psychiatry.

[20] Kessler R.C., Keyes C.L.M., Goodman S.H. (2006). The epidemiology of depression among women. Women and Depression.

[21] Spitzer R.L., Kroenke K., Williams J.B., Löwe B. (2006). A brief measure for assessing generalized anxiety disorder: The GAD-7. Arch. Intern. Med..

[22] Clements-Nolle C., Marx R., Guzman R., Katz M. (2001). HIV prevalence, risk behaviors, health care use and mental health status of transgender persons: Implications for public health intervention. Am. J. Public Health.

[23] Nemoto T., Bodeker B., Iwamoto M. (2011). Social support, exposure to violence and transphobia: Correlates of depression among male-to female transgender women with a history of sex work. Am. J. Public Health.

[24] Bockting W., Coleman E., Deutsch M.B., Guillamon A., Meyer I., Meyer W., Reisner S., Sevelius J., Ettner R. (2016). Adult development and quality of life of transgender and gender nonconforming people. Curr. Opin. Endocrinol. Diabetes Obes..

[25] Koken J.A., Bimbi D.S., Parsons J.T. (2009). Experiences of familial acceptance–rejection among transwomen of color. J. Fam. Psychol..

[26] Erich S., Tittsworth J., Kerstein A.S. (2010). An examination and comparison of transsexuals of color and their white counterparts regarding personal well-being and support networks. J. GLBT Fam. Stud..

[27] Pruzinski T., Cash T.F., Pruzinski T. (1990). Psychopatology of body experience: Expanded perspectives. Body Images: Development, Deviance and Change.

[28] White J.M., Reisner S.L. (2016). A systematic review of the effects of hormone therapy on psychological functioning and quality of life in transgender individuals. Transgend. Health.

[29] McClelland S.I. (2010). Intimate justice: A critical analysis of sexual satisfaction. Soc. Personal. Psychol. Compass.

[30] Devor H. (1997). FTM: Female-to-Male Transsexuals in Society.

[31] Bowlby J. (1969). Attachment and Loss.

[32] Leary M.R., Tambor E.S., Terdal S.K., Downs D.L. (1995). Self-esteem as an interpersonal monitor: The sociometer hypothesis. J. Personal. Soc. Psychol..

[33] Antonovsky A. (1988). Unraveling the Mystery of Health. How People Manage Stress and Stay Well.

[34] McCubbin M.A., McCubbin H.I. (1989). Theoretical orientations to family stress and coping. Treating Stress in Families.

[35] Orth U., Robins R.W., Meier L.L. (2009). Disentangling the effects of low self-esteem and stressful events on depression: Findings from three longitudinal studies. J. Personal. Soc. Psychol..

[36] Crocker J., Park L.E. (2004). The costly pursuit of self-esteem. Psychol. Bull..

[37] Bouman W.P., Claes L., Brewin N., Crawford J.R., Millet N., Fernandez-Aranda F., Arcelus J. (2017). Transgender and anxiety: A comparative study between transgender people and the general population. Int. J. Transgend..

[38] Witcomb G.L., Bouman W.P., Claes L., Brewin N., Crawford J.R., Arcelus J. (2018). Levels of depression in transgender people and its predictors: Results of a large matched control study with transgender people accessing clinical services. J. Affect. Disord..

[39] Pruzinsky T., Cash T.F., Cash T.F., Pruzinsky T. (1990). Integrative themes in body-image development, deviance and change. Body Images: Development, Deviance and Change.

[40] Beiter R., Nash R., McCrady M., Rhoades D., Linscomb M., Clarahan M., Sammut S. (2015). The prevalence and correlates of depression, anxiety and stress in a sample of college students. J. Affect. Disord..

[41] Röder M., Barkmann C., Richter-Appelt H., Schulte-Markwort M., Ravens-Sieberer U., Becker I. (2018). Health-related quality of life in transgender adolescents: Associations with body image and emotional and behavioral problems. Int. J. Transgend..

[42] Martinsen K.D., Kendall P.C., Stark K., Neumer S.P. (2016). Prevention of anxiety and depression in children: Acceptability and feasibility of the transdiagnostic EMOTION program. Cogn. Behav. Pract..

[43] Grossman A.H., D’augelli A.R., Frank J.A. (2011). Aspects of psychological resilience among transgender youth. J. LGBT Youth.

[44] Hawkley L.C., Burleson M.H., Berntson G.G., Cacioppo J.T. (2003). Loneliness in everyday life: Cardiovascular activity, psychosocial context and health behaviors. J. Personal. Soc. Psychol..

[45] DiTommaso E., Brannen C., Best L.A. (2004). Measurement and validity characteristics of the short version of the social and emotional loneliness scale for adults. Educ. Psychol. Meas..

[46] Klein A., Golub S.A. (2016). Family rejection as a predictor of suicide attempts and substance misuse among transgender and gender nonconforming adults. LGBT Health.

[47] López F. (2008). Necesidades en la infancia y en la adolescencia. Respuesta Familiar, Escolar y Social.

[48] Zimmer D. (1987). Does marital therapy enhance the effectiveness of treatment for sexual dysfunction?. J. Sex Marital Ther..

[49] Carcedo R.J., Perlman D., López F., Orgaz M.B., Toth K., Fernández-Rouco N. (2008). Men and women in the same prison: Interpersonal needs and psychological health of prison inmates. Int. J. Offender Ther. Comp. Criminol..

[50] Carcedo R.J., Perlman D., Orgaz M.B., López F., Fernández-Rouco N., Faldowski R.A. (2011). Heterosexual romantic relationships inside of prison: Partner status as predictor of loneliness, sexual satisfaction and quality of life. Int. J. Offender Ther. Comp. Criminol..

[51] Carcedo R.J., Perlman D., López F., Orgaz M.B. (2012). Heterosexual romantic relationships, interpersonal needs and quality of life in prison. Span. J. Psychol..

[52] Carcedo R.J., Perlman D., López F., Orgaz M.B., Fernández-Rouco N. (2015). The relationship between sexual satisfaction and psychological health of prison inmates. Prison J..

[53] Davis S.A., Meier S. (2014). Effects of testosterone treatment and chest reconstruction surgery on mental health and sexuality in female-to-male transgender people. Int. J. Sex Health.

[54] Timmins L., Rimes K.A., Rahman Q. (2017). Minority stressors and psychological distress in transgender individuals. Psychol. Sex. Orientat. Gend. Divers..

[55] Goodman L.A. (1961). Snowball sampling. Ann. Math. Stat..

[56] CSIC (2011). Código de Buenas Prácticas Científicas del CSIC. https://www.cnb.csic.es/documents/CBP_CSIC.pdf.

[57] Fitts W., Warren W. (1996). Tennessee Self-Concept Scale (2^ª^ Ed.).

[58] Fitts W.H. (1964). The Tennessee Self-Concept Scale, Mind Over Matter.

[59] Lindgren T., Pauly I. (1975). A body image scale for evaluating transsexuals. Arch. Sex. Behav..

[60] Basabe N., Valdoseda M., Páez D., Páez D. (1993). Memoria afectiva, salud, formas de afrontamiento y soporte social. Salud, Expresión y Represión Social de las Emociones.

[61] Lazarus R., Folkman S. (1986). Estrés y Procesos Cognitivos.

[62] Derogatis L.R., Lipman R.S., Covi L. (1973). SCL-90: An outpatient psychiatric rating scale—Preliminary report. Psychopharmacol. Bull..

[63] Erdfelder E., Faul F., Buchner A. (1996). GPOWER: A general power analysis program. Behav. Res. Methods Instrum. Comput..

[64] Daryanto A. (2018). Heteroskedasticity Test for SPSS (2nd Version). https://sites.google.com/site/ahmaddaryanto/scripts/Heterogeneity-test.

[65] Hoffman B.R. (2014). The interaction of drug use, sex work and HIV among transgender women. Subst. Use Misuse.

[66] Sevelius J.M., Reznick O.G., Hart S.L., Schwarcz S. (2009). Informing interventions: The importance of contextual factors in the prediction of sexual risk behaviors among transgender women. AIDS Educ. Prev..

[67] Sirin S.R., McCreary D.R., Mahalik J.R. (2004). Differential reactions to men and women’s gender role transgressions: Perceptions of social status, sexual orientation and value dissimilarity. J. Mens. Stud..

[68] Maser J.D., Cloninger C.R. (1990). Comorbidity of anxiety and mood disorders: Introduction and overview. Comorbidity of Mood and Anxiety Disorders.

[69] Endler N.S., Macrodimitris S.D., Kocovski N.L. (2003). Anxiety and Depression: Congruent, Separate or Both?. J. Appl. Biobehav. Res..

[70] Endler N.S., Cox B.J., Parker J.D., Bagby R.M. (1992). Self-reports of depression and state-trait anxiety: Evidence for differential assessment. J. Pers. Soc. Psychol..

[71] Meyer I.H. (1995). Minority stress and mental health in gay men. J. Health Soc. Behav..

[72] DiTommaso E., Spinner B. (1997). Social and emotional loneliness: A re-examination of Weiss’ typology of loneliness. Personal. Individ. Differ..

[73] Nangle D.W., Erdley C.A., Newman J.E., Mason C.A., Carpenter E.M. (2003). Popularity, friendship quantity and friendship quality: Interactive influences on children’s loneliness and depression. J. Clin. Child. Adolesc. Psychol..

[74] Prince M.J., Harwood R.H., Blizard R.A., Thomas A., Mann A.H. (1997). Social support deficits, loneliness and life events as risk factors for depression in old age. The Gospel Oak Project VI. Psychol. Med..

[75] Segrin C., Powell H.L., Givertz M., Brackin A. (2003). Symptoms of depression, relational quality and loneliness in dating relationships. Pers. Relatsh..

[76] Dear G.E., Thomson D.M., Hall G.J., Hall K., Kosky R.J., Eshkevarky S., Hassan R., Goldney R. (1998). Self-inflicted injury and coping behaviours in prison. Suicide Prevention: The Global Context.

[77] Mustanski B.S., Garofalo R., Emerson E.M. (2010). Mental health disorders, psychological distress and suicidality in a diverse sample of lesbian, gay, bisexual and transgender youths. Am. J. Public Health.

